# Conceptualizing Sexual Wellbeing: A Qualitative Investigation to Inform Development of a Measure (Natsal-SW)

**DOI:** 10.1080/00224499.2024.2326933

**Published:** 2024-03-22

**Authors:** R. Lewis, R. Bosó Pérez, K.J. Maxwell, D. Reid, W. Macdowall, C. Bonell, J.D. Fortenberry, C.H. Mercer, P. Sonnenberg, K.R. Mitchell

**Affiliations:** a MRC/CSO Social and Public Health Sciences Unit, University of Glasgow; b Research Centre for Health, Glasgow Caledonian University; c Institute for Global Health, University College London; d Faculty of Public Health & Policy, London School of Hygiene & Tropical Medicine; e Department of Pediatrics, Indiana University School of Medicine

## Abstract

Despite increasing scientific and policy interest in sexual wellbeing, it remains poorly conceptualized. Many studies purporting to measure it instead measure related but distinct concepts, such as sexual satisfaction. This lack of conceptual clarity impedes understanding, measuring, and improving sexual wellbeing. We present qualitative research from multi-stage, mixed-methods work to develop a new measure of sexual wellbeing (Natsal-SW) for the fourth British National Survey of Sexual Attitudes & Lifestyles. Literature review and discussion generated a conceptual framework with seven proposed domains: respect, self-esteem, comfort, self-determination, safety and security, forgiveness, and resilience. Semi-structured interviews with 40 adults aged 18–64 then explored whether and how these domains aligned with participants’ own understandings, experiences, and language of sexual wellbeing. Data were analyzed thematically. Participants conceptualized sexual wellbeing as distinct from sexual satisfaction and sexual health and as multidimensional, dynamic, and socially and structurally influenced. All seven proposed domains resonated with accounts of sexual wellbeing as a general construct. The personal salience of different domains and their dimensions varied between individuals (especially by gender and sexual orientation) and fluctuated individually over time. This study clarifies dimensions of domains that participants considered important, providing an empirical basis to inform development of a new measure of sexual wellbeing.

## Introduction

Within the field of sexual health, there is a long-standing recognition that holistic and positive perspectives on sexuality matter for public health (Ford et al., 2019; Gruskin & Kismödi, 2020; Laumann et al., 2006; Wellings & Johnson, 2013). Sexual wellbeing is gaining traction as a concept that could promote this agenda (Lorimer et al., 2023). However, increasing reference to sexual wellbeing in research and policy (e.g. Department of Health, 2013, 2015; Scottish Government, 2015) has not been accompanied by increasing clarity in definition and measurement (Martin & Woodgate, 2020; Sundgren et al., 2022).

This paper is part of a broader project to conceptualize and operationalize sexual wellbeing for public health research and practice, including the development of a brief measure for population surveys, described elsewhere (Mitchell et al., 2023). We have previously argued that sexual wellbeing can be conceptualized as one of four overlapping – yet distinct – pillars for a comprehensive public health perspective on sexuality, the other three being sexual health (encompassing STI prevention and management, fertility management, sexual violence prevention, sexual function, desire, and arousal), sexual justice (sexual rights, citizenship, and positive practice), and sexual pleasure (event-related, person-related) (Mitchell et al., 2021). We assert that sexual wellbeing demands recognition as a public health outcome in its own right, and propose a conceptual framework for this construct comprising seven domains, namely: sexual respect; sexual self-esteem; comfort with sexuality; self-determination in one’s sexual life; sexual safety and security; forgiveness of past sexual experiences; and resilience in relation to sexual experience.

Here, we explore how public understandings of sexual wellbeing align with our conceptualization. The question then follows: how has sexual wellbeing been conceptualized in scholarly literature so far?

### Conceptualizing and Measuring Sexual Wellbeing

In 2007, a World Health Organization/United Nations Population Fund working group on sexual health indicators failed to reach an agreement on what “sexual wellbeing” meant and how to measure it, concluding that it was perhaps best defined as “self-perceived sexual health” (World Health Organization, 2010, p. 4). Since then, there have been many attempts to define and measure sexual wellbeing as a distinct construct (e.g. Contreras et al., 2016; Foster & Byers, 2013; Muise et al., 2010; Pearlman-Avnion et al., 2017). Collectively, 694 R. LEWIS ET AL. this field of endeavor is characterized by a lack of consensus on the definition and conceptualization of sexual wellbeing, as highlighted by two recent reviews. In a review of 162 studies in which sexual wellbeing was either directly measured or described as an outcome, Lorimer et al. (2019) found only 10 of the studies explicitly defined sexual wellbeing. Similarly, building on the work of Lorimer and colleagues, a recent review of 74 psychometric measures identified by searching for the term “sexual wellbeing,” found less than a third of studies defined sexual wellbeing (Sundgren et al., 2022). Authors of both reviews argued that this lack of explicit definition and dearth of clear conceptualization of sexual wellbeing as a construct underpins divergence in how it is operationalized.

The review by Sundgren et al. (2022) found that sexual function and sexual satisfaction were the most frequently observed dimensions across 74 measures of sexual wellbeing. In some cases, sexual wellbeing has largely been used as a synonym for sexual satisfaction, as in Laumann et al.’s (2006) assessment of sexual wellbeing among older adults, based on indications of relational satisfaction (emotional and physical), satisfaction with sexual health and function, alongside importance of sex in one’s life. Other efforts to operationalize sexual wellbeing have included items on sexual satisfaction and function alongside a wider range of dimensions, including the frequency of sexual activity. For instance, in their study of older adults, Štulhofer et al. (2019) employed a measure of sexual wellbeing that included items on sexual satisfaction and distress over sexual function, alongside items on frequency of cuddling and caressing, emotional intimacy during sex, and sexual compatibility. Another recent measure, the Short Sexual Well-being Scale (SSWBS), also focused on function and satisfaction (items on absence of sexual distress, physical sexual satisfaction, emotional sexual fulfillment), alongside items on sexual frequency and ability to realize one’s sexual fantasies (Gerymski, 2021). However, Sundgren et al. (2022) questioned the validity of measures used to assess sexual wellbeing that combine measurement of a range of different dimensions or indicators of sexual wellbeing (such as sexual satisfaction and function) as opposed to capturing an overarching construct. They argued that the struggle to differentiate the construct of sexual wellbeing from its potential correlates is exacerbated by the lack of theory-driven conceptualization and careful definition.

Limited attention to social and structural factors shaping sexual wellbeing has been identified as a further shortcoming within existing measurement. The review by Lorimer et al. (2019) drew upon a socioecological perspective to categorize the 59 dimensions of sexual wellbeing that were studied into three domains. They found that most dimensions pertained to the individual cognitive-affect domain (141 studies), with far fewer relating to the interpersonal domain (52 studies), and very little representation of the sociocultural domain (only 10 studies). The underrepresentation of interpersonal and sociocultural dimensions in efforts to measure sexual wellbeing is antithetical to longstanding literature evidencing how sexuality is “structured by sociodemographic, relational, sociocultural, and structural forces” (Higgins et al., 2022).

Moreover, items for measures of sexual wellbeing are commonly developed based solely on researchers’ understandings (Sundgren et al., 2022), rather than informed by qualitative evidence about how these concepts are understood and experienced among the general population, raising important epistemological considerations about whose voices are heard within measure development (Lorimer et al., 2023). The extent to which conceptualizations of sexual wellbeing among those engaged in scholarly research align with public understandings remains underexplored.

In summary, current efforts to measure sexual wellbeing are characterized by lack of consensus over how sexual wellbeing should be conceptualized and defined, limited theorization of sexual wellbeing as a construct, scant attention to capturing interpersonal and structural dimensions of sexual wellbeing, and a proliferation of measures based solely upon researchers’ conceptualizations, rather than being grounded in public understandings.

### A New Conceptual Framework of Sexual Wellbeing

We previously proposed a conceptual framework of sexual wellbeing comprising seven domains, namely: sexual respect; sexual self-esteem; comfort with sexuality; sexual self-determination; sexual safety and security; forgiveness of past sexual experiences; and resilience in relation to sexual experience (Mitchell et al., 2021). These proposed domains represent facets of psychological and emotional wellbeing distinct from – but pertinent to – sexuality and sexual health. Building on calls for greater attention to interpersonal and sociocultural elements of sexual wellbeing (Lorimer et al., 2019, 2023; Sundgren et al., 2022), our framework conceptualizes each of the seven domains as dynamically shaped through an interplay of individual, interpersonal, and sociocultural factors, and through interactions between domains.

Our conceptual framework was generated through a literature review, alongside structured discussion between authors. Given the limitations of the sexual wellbeing literature described above, we adopted a broader perspective, seeking to apply theoretical work on wellbeing (including from positive psychology) to the sexuality domain. We researched and discussed domains established within the literature (e.g. sexual self-esteem), as well as those that are less established but theoretically convincing (e.g. sexual forgiveness). We were orientated by our public health perspective, specifying that sexual wellbeing should be distinct from sexual health, sexual function, sexual satisfaction and pleasure; able to capture people’s experiences regardless of their age, gender, sexual identity, sexual activity, or partnership status; amenable to change; and focused on both past experiences and near-future expectations. We included a near-future time reference to include a predictive capacity, in addition to evaluative functions, of sexual wellbeing. A key test of this conceptual work lies in its relevance to lived experiences of sexuality.

**Figure 1:**
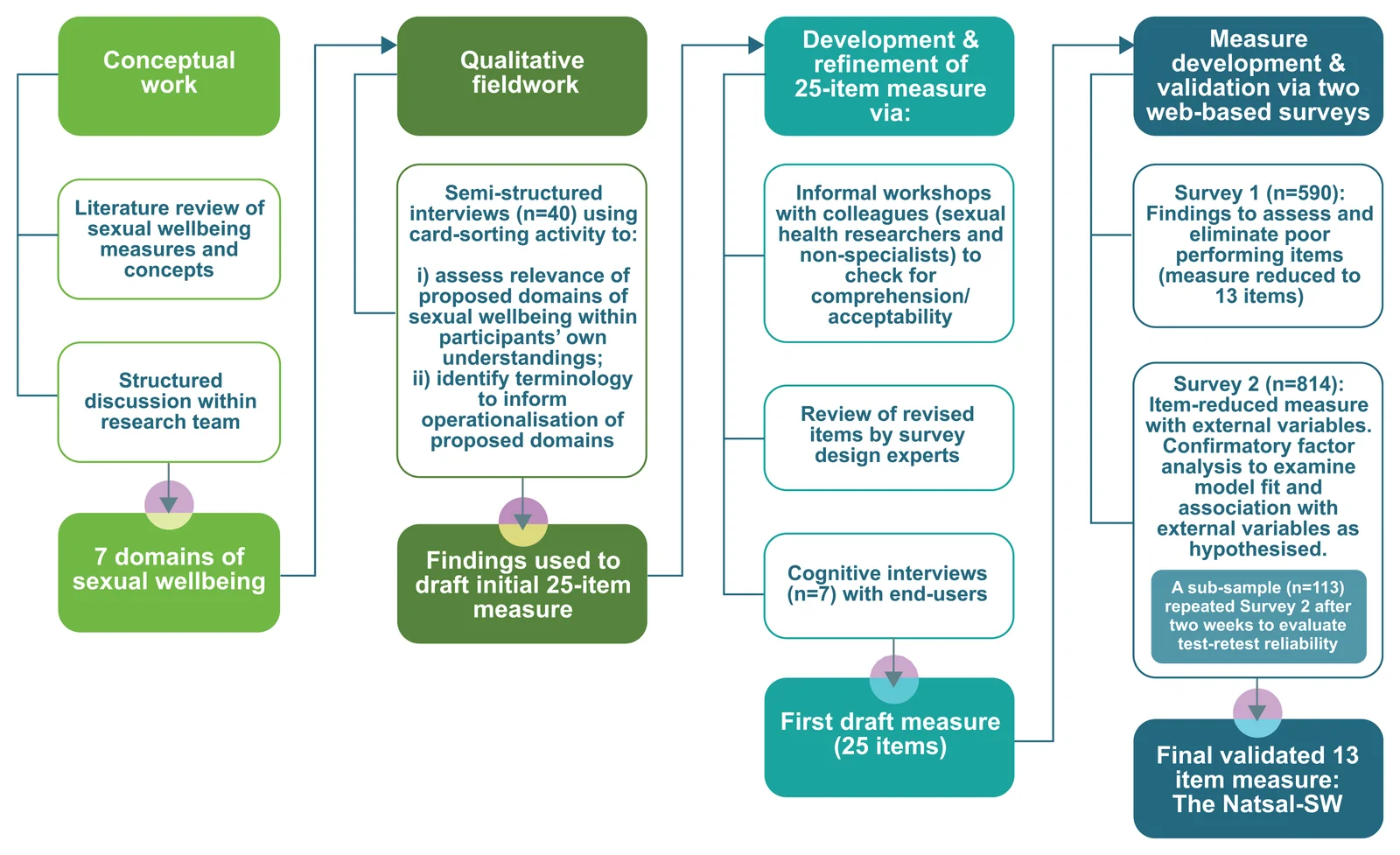
Multi-stage, mixed methods process to develop a new 13-item measure of sexual wellbeing: the Natsal-SW.

This paper describes the qualitative component of a multistage, mixed-methods program of development work (see Figure 1) to design a brief measure of sexual wellbeing for the fourth British National Survey of Sexual Attitudes and Lifestyles (Natsal-4). Natsal is a national probability sample survey conducted approximately decennially since 1990. As shown in Figure 1, the qualitative study reported here followed work to conceptualize sexual wellbeing which generated seven theoretically proposed domains (Mitchell et al., 2021). The purpose of the qualitative study was to explore our initial conceptualization of sexual wellbeing, guided by two research questions:

**RQ1:** How do participants describe sexual wellbeing?

**RQ2:** To what extent do seven theoretically proposed domains of sexual wellbeing resonate with participants’ understandings and experiences of sexual wellbeing?

This qualitative work informed the development and validation of the Natsal-SW, a 13-item measure, reported in a partner paper (Mitchell et al., 2023).

## Method

We conducted 40 in-person semi-structured interviews to inform the development of the Natsal-SW as well as the development of new questionnaire items for Natsal-4 on another novel topic: the role of digital technologies in sexuality. Twenty of these interviews focused primarily on sexual wellbeing (i.e. approximately two-thirds of the interview time spent discussing sexual wellbeing) with a secondary focus on digital technologies, while in the other 20, the focus was reversed (i.e. approximately one-third of the interview time spent discussing sexual wellbeing). Our qualitative work informing the development of new questions on digital technologies is reported elsewhere (Macdowall et al., 2023; Reid et al., 2022). Ethical approval was granted by the University of Glasgow MVLS Ethics Committee (reference 200190039) and LSHTM’s Research Ethics Committee (reference 1704626/4/2019).

### Participants

Six researchers (diverse in age, gender, sexual orientation, and nationality) designed and conducted interviews with adults aged 18–64 (see Table 1 for participant characteristics) in May and June 2019. A market research recruitment agency (propeller-research.co.uk) recruited participants from among adult residents of Britain via media advertisement, contacting those registered with the company as available for interview. Our approach to sampling aimed to ensure variation in terms of age, gender, sexual orientation, ethnicity, current relationship status, and area-level deprivation (based on postcode). The recruitment agency emailed potential participants, using quota sampling, and sent the research team the e-mail addresses of those who consented to be contacted. Care was taken to ensure a geographic spread of interviewees across England, Scotland, and Wales.

### Procedure

Interviews took place in participants’ homes or another venue of their choice (e.g. university offices). Only the participant and interviewer were present in all but one interview (in which a participant requested a friend’s presence). Interviews lasted 21–78 min (mean 60 min) and were audio-recorded with participants’ consent. After each interview, researchers completed field notes summarizing and reflecting on the interaction. Participants received £40 in appreciation of their time.

Interviews were semi-structured, using a topic guide (see Online Supplemental File) that broadly organized discussion about sexual wellbeing into two parts. The first part of the interview used open questions to elicit and explore participants’ own understandings, experiences, and terminologies relating to sexual wellbeing. Topics included feelings about their sex life, encompassing what constitutes a “good sex life” and reflection on any change in their sex life over time; meanings of wellbeing in general; meanings of sexual wellbeing; the range of criteria used to assess whether one has sexual wellbeing (both for them personally and in general); reflection on sexual wellbeing over their life course (including views on causes of stability or fluctuation in sexual wellbeing); and perceived facilitators and barriers to sexual wellbeing.

Once participants’ own initial meanings of sexual wellbeing had been elicited, in the second part of the interview we then introduced concepts relating to each of the proposed domains of sexual wellbeing in order to assess the extent to which these were salient to participants’ own understandings and experiences. At no time did we present interviewees with the seven domains – rather, participants were given 20 flash cards, each with a statement reflecting researchers’ initial attempts to operationalize key dimensions of the seven proposed domains of sexual wellbeing, based on literature review and discussion (see list of statements in Table 2). Participants were invited to read the cards and sort them into three piles indicating the importance that the statements had to their own sense of sexual wellbeing (“Very important,” “Quite important,” “Not that important”). Participants were also invited to add and categorize any statements of their own if they felt something important to their own understanding was not captured in the original set of cards. Once participants had sorted the cards, the interviewer probed participants’ rationale for their categorizations, and invited further discussion of any statements about which they were unclear. We intended that discussions about the cards would i) elucidate whether the proposed domains resonated with participants’ understandings and experiences of sexual wellbeing; ii) generate further understanding of the domains and their interconnections; and iii) offer examples of vocabulary to assist with item development within the measure.

Several steps were taken to securely manage data and protect participants’ identity. Interviews were transcribed verbatim by a professional company, with agreed procedures for secure and confidential data transfer. Identifying details (e.g. names, locations) were then removed from transcripts by researchers, and participants were assigned a unique participant identification number. De-identified transcripts were stored separately from interview audio files. A document recording participants’ identifiers and linked to participant identification numbers was also stored separately. Access to all files was restricted to team members only, using different password protection for different files. De-identified transcripts were entered into a qualitative data management software program (NVivo v.12) to facilitate coding and analysis.

### Data Analysis

We conducted a thematic analysis of the data. As discussed by Braun and Clarke (2019, 2021a, 2021b) thematic analysis is best characterized as a spectrum of approaches where analysts have choices between a range of options; what matters is design coherence – or “fit” – between analytic approach, researchers’ epistemological assumptions, and the research goals. Our approach was rooted within a constructivist and interpretivist paradigm, whereby we viewed meanings of sexual wellbeing as multiple, contingent, and generated through social interaction. Moreover, researchers’ reflexivity and subjectivity was viewed as a resource, rather than a threat, to knowledge production (Braun & Clarke, 2019). To address our dual analytic goals (to understand participants’ own meanings of sexual wellbeing, and to assess the salience of our proposed domains within these understandings), our thematic analysis was an iterative process involving both inductive and deductive elements. We familiarized ourselves with the data through reading full interview transcripts, alongside the accompanying fieldnotes from the interviewer. Two researchers (RL and RBP) generated an initial codebook through separately open coding of a sub-sample of transcripts, with regular discussion with each other, and also among the wider team about new codes and their place in the evolving codebook. The codebook included inductively generated codes reflecting participants’ meanings and terminology (see Online Supplemental File for codes on meanings of sexual wellbeing). The seven proposed domains of sexual wellbeing were a priori codes, with our application of these codes focused on identifying all instances of text within transcripts which related to each of the domains, as assessed by the research team. For example, we used the code “self-determination” to code participant talk that related to our proposed conceptualization of this domain even if participants did not explicitly use the term “self-determination” in their accounts. One researcher (RBP) coded the majority of the rest of the transcripts (with contributions from RL), generating new codes to capture meanings not included in the initial codebook.

Through deep engagement with the data, we generated themes to capture meanings of sexual wellbeing as an overarching concept (e.g. “sexual wellbeing as work-in-progress”; “sexual wellbeing as distinct from sexual health”). In line with our analytic goal to assess the salience of our proposed domains, another strand of analysis focused on developing domain summaries capturing the diversity of meaning in relation to each proposed domain (Braun & Clarke, 2019). To do this, we used charting to summarize meanings, and to gain an initial sense of variations in participants’ understandings and experiences pertaining to each domain. We then developed analytic memos, first for each participant (informed by review both of transcripts and researchers’ field notes from interviews), and then for each domain, which included identifying dominant and minority views and experiences elating to each domain, and mapping patterns in the salience of domains in relation to participants’ identities and experiences. This process was informed by reviewing participants’ rankings and subsequent discussion of the importance of items on statement cards (see Table 3). Finally, we mapped interconnections between domains via axial coding, including within a detailed case analysis of 20 participants (those where interviews had primarily focused on sexual wellbeing) to deepen our understanding of sexual wellbeing as a multidimensional, dynamic, and complex construct.

## Results

Below we present a synthesis of participants’ accounts, first focusing on how sexual wellbeing was conceptualized as an overarching concept, and then exploring if and how each of the seven proposed domains within our conceptualization of sexual wellbeing resonated with participants’ understandings and experiences. We then describe interactions between the domains of sexual wellbeing, using two case examples to illustrate how these interactions play out in the context of individual lives, and are dynamically shaped through social and structural elements. Throughout our synthesis, illustrative extracts from interviews are presented alongside sociodemographic characteristics (gender, sexual orientation, age, ethnicity, and relationship status) which participants self-identified via a screening questionnaire during recruitment to the study. The ethnicity categories reported reflect participants’ endorsement of their ethnicity in relation to ethnic groups used within the Census for England and Wales.^1^

### What is Sexual Wellbeing? Accounts of Sexual Wellbeing as an Overall Concept

When asked what sexual wellbeing meant to them, participants often initially commented that it was challenging to articulate, indicating that the concept is not well defined within everyday discourse, or routinely reflected upon; for example: *Like, erm, [pause], I guess* it’s *like how [pause], yeah, I* don’t, *I* don’t *really know* …” (Heterosexual woman, aged 55, Black, married), and *“I think to be* honest like *sexual wellbeing* it’s *not something* that, *that word and the connotations I* don’t *really think about it that often* …” (Heterosexual man, aged 22, White, single). Yet, as interviewees started reflecting on their sexual experiences in greater depth, they proceeded to offer more detailed accounts of what sexual wellbeing meant in the context of their lives.

Participants commonly distinguished sexual wellbeing from sexual health, the latter of which was routinely framed in terms of prevention of sexually transmitted infections:

I think sexual health is, is different to sexual wellbeing. [Int: Ah okay. Why’s that?] Um, I suppose wellbeing is focused on, you know, what makes you happy sexually, um, are you happy sexually? Um, sexual health is, is, uh, all about, um, protection, you know, use of condoms etc. […] So I, although they’re sort of linked I don’t, I see them separately I would say. (Gay man, aged 22, White, in a relationship)

A majority view of sexual wellbeing positioned it as a multidimensional concept encompassing interplay between individual and relational elements which contribute to the quality of one’s sexual experiences and feelings about one’s sexual self. The depth and complexity of these multiple dimensions were evident in the following extracts:

I think sexual wellbeing is a feeling of wellness in yourself, in being able to talk with your partner, not feel ridiculed in whatever way. Trust is a core part of that wellbeing but also, as well as obviously with your partner you have to think of yourself as well and that’s also to do with probably your health, so again in the mind and the body, so it’s getting checked [i.e. tested for STIs] for your own sake, especially if you are sexually active with many people … (Heterosexual man, aged 29, White, single)

I see [sexual] wellbeing as being quite a broad thing, like a bit distant, it encompasses like safety, enjoyment […] I guess some people, you know, in their life have sex just for the sake of pleasing a partner and then, you know, it’s done for the week or something like that […] I guess with sexual wellbeing, I maybe think about it more now maybe as a connection between two people, versus just doing it to please somebody else or just, you know, just for yourself, I guess. (Heterosexual man, aged 32, White, in a relationship)

At the individual level, participants described affective understandings of sexual wellbeing relating to one’s sense of ease and comfort regarding sex, satisfaction with the type and frequency of the sex one has, and confidence to be able to understand and articulate one’s sexual desires. A small minority of participants also described aspects of sexual function within their talk about sexual wellbeing, including erectile function and being physically fit enough to be able to have the type of sex that was desired. Relational elements that featured prominently within participants’ accounts of sexual wellbeing included the ability to pursue satisfying sexual relationships with others, which was described as requiring abilities to understand other people’s sexual desire and interpret a situation as consensual, express one’s own sexual likes and needs, and navigate those with a partner, alongside an ability to communicate one’s own boundaries and accept those of a sexual partner.

Sociocultural elements were present in accounts of sexual wellbeing, though featured less heavily than individual and interpersonal factors. The most commonly raised elements were social norms that inhibit sexual wellbeing by perpetuating stigma, exclusion, or violence on the basis of one’s physical appearance (e.g. fatphobia), reproductive choices (e.g. to have an abortion), or sexual or gender identity:

**Int: Um, what does sexual wellbeing mean to you?**

[…] like checking that people are okay within, um… Like whether they’re gay or whether they’re straight and information about like various things, like sexual diseases and that kind of stuff… [Pause] Um, so like if people choose to be, whatever they want to be, gay, straight, I don’t know, transgender, you know, that kind of stuff, that um, they’re not being like targeted maybe, so like their wellbeing is looked after. (Heterosexual woman, aged 29, White, married)

Participants clearly identified sexual wellbeing as distinct from sexual pleasure and satisfaction; for example:

I think it’s [sexual wellbeing] bigger [than sexual satisfaction] because just your whole wellbeing is more important than just being satisfied so to speak. Like you can be satisfied with what, I don’t know, some people can be 15 minutes, I don’t know, like you can be satisfied for a long, short period, however it may be for you at that time, but your wellbeing can be affected in the long run because just your whole wellbeing is more important than just being satisfied so to speak.(Heterosexual woman, aged 24, Mixed ethnicity, single)

As in the extract above, sexual satisfaction was commonly framed by participants as a transitory experience during and immediately after sex. It was also often discussed synonymously with sexual pleasure. In contrast, sexual wellbeing was conveyed as a broader sense of fulfillment with one’s sexual life. Yet satisfaction and wellbeing were also seen as intimately intertwined, with the relationship being bidirectional. On the one hand, sexual wellbeing was described as leading to satisfying sex: “*as long as happiness is there, ‘cause obviously if it’s not there it’s not pleasurable*” (Gay man, aged 25, White, single). On the other hand, sexual satisfaction was seen as contributing to broader sexual wellbeing, with the threat of its absence seen as having a negative impact; as one person noted: “ … *for me satisfaction would play a part in sexual wellbeing because I wouldn’t want to be dissatisfied, but equally I wouldn’t want my partner to be dissatisfied because at the end of the day that would come back out and then would affect other parts of my kind of wellbeing*” (Heterosexual man, aged 32, White, in a relationship). In general, accounts conveyed the complex interconnection between sexual satisfaction, pleasure, and sexual wellbeing. Sexual wellbeing, while foundational to sexual pleasure and satisfaction, was seen as conceptually distinct:

I couldn’t say my wellbeing and pleasure are equal, they may be linked but I wouldn’t say they’re the same […] Yeah, obviously, you know, if you are happy then you will likely have more pleasure which increases your wellbeing but I wouldn’t say that if you increase your wellbeing that increases your pleasure necessarily. Because you can feel better about yourself but you don’t necessarily… I’m not currently sexually active with anyone and I don’t feel the best about myself… So my wellbeing probably is not as high as it once was but I certainly do not… That does not detract from the pleasure of what sex would be. (Heterosexual man, aged 29, White, single)

Reflecting on sexual wellbeing across their life course, participants identified a range of factors they viewed as promoting sexual wellbeing, including confidence and knowledge accrued through experience, increased understanding and maturity, deeper knowledge and understanding of others within a long-term partnership, and shifting cultural norms (e.g. those promoting inclusion and affirmation of LGBTQ+ identities and experiences). Nevertheless, participants’ accounts did not indicate a smooth and linear progression toward better sexual wellbeing with age, but rather described sexual wellbeing as fluctuating in the context of different life events (including illness, parenting, relationship breakdown), and in relation to the evolving socio-sexual landscape. The process of developing and sustaining sexual wellbeing was conveyed as a constant work-in-progress. As such, participants’ accounts affirmed our a priori view of sexual wellbeing as more than just an appraisal of specific sexual experiences or situations, but rather as a summation of appraisals of broader experiences and near-future expectations related to sexuality that were shaped by and through accumulated interactions over time.

### Meanings and Salience of the Proposed Domains of Sexual Wellbeing within Participants’ Accounts

Below, we present a synthesis of participants’ accounts relating to each of the proposed domains of sexual wellbeing. The synthesis is organized per domain in response to our objective to assess whether and how our seven theoretically proposed domains were characterized within participants’ accounts of their understandings and experiences. As our goal was to inform the development of a measure fit for purpose within population surveys (Mitchell et al., 2023), our analysis focused on assessing variations in meanings and salience of each domain, and identifying commonly used terminology that could support item development within the Natsal-SW measure. Key learning relating to these aspects is summarized in the final column of Table 2.

A key point to note is that the data presented in these sections reflect participants’ talk across the entirety of the interview, and not just once the card-sorting activity commenced. As such, across the sample, dimensions relating to all seven domains were spontaneously raised by participants in the first part of the interviews, before they were presented with the statements in the card-sorting activity which reflected the proposed domains.

### Sexual Respect

The concept of sexual respect – which we defined as perception of positive regard by others for one’s sexual personhood – resonated strongly within participants’ accounts of sexual wellbeing. Across our sample, participants prioritized feeling respected by sexual partners and friends over unknown others in wider society (i.e. the generalized other). In particular, feeling valued as a sexual partner – whether short or long term – was a necessary precursor to “good sex” for some:

… as long as you’ve spent that night with them you’ve bonded over something or you’ve enjoyed their company, you’ve had good conversations or in-depth conversations that you’re kind of, at least you know the person on a sort of level and … you’re valued by them, I think that is very important. (Heterosexual man, aged 22, White, single)

In addition to sexual partners, prioritizing the views of others close to them allowed individuals to craft social networks that supported their sexual wellbeing, even in cases where wider societal acceptance of their sexual personhood may be questioned, for example:

I really don’t care if you [i.e. generalised “you”] accept me or not […] I know that people around me share the same values, because they wouldn’t be around me otherwise. (Lesbian woman, aged 32, White, married)

You’re never going to please all of society […] as long as you’re comfortable with who you are, you’ve got some people around you who are comfortable with who you are, you can be who you are, who cares beyond that what others think? (Heterosexual man, aged 32, White, cohabiting)

In contrast, several participants (mainly women) described how being held in low regard by others negatively affected their ability to communicate and uphold personal boundaries within sexual interactions and relationships. For example, one woman implicitly linked lack of sexual respect to sexual violence and coercion, recounting multiple men’s fatphobic comments and disregard of her sexual personhood as contributing to her response to abuse (emotional, physical, sexual) within a former long-term relationship: “*my son’s dad technically raped me [*…*] I didn’t really tell anybody because I thought nobody’s going to give a fuck anyway so I’ll just keep it to myself*.” She contrasted these experiences to those with her current relationship, which she characterized as involving a high degree of mutual regard for understanding and upholding each other’s sexual boundaries:

I guess because we’ve both been through quite traumatic sexual experiences in a sense that we know what we want from each other but we don’t, like before we go do something we’ll ask us if it’s okay to do that, it’s erm, like out of courtesy … like I might want to do this but you might not want to […] but that is a part of sexual wellbeing, like you need to have that respect or else there’s just no boundaries. (Bisexual woman, aged 25, White, in a relationship)

Despite participants stating that they placed greater value on feeling respected by sexual partners and close connections, nevertheless wider social acceptance of one’s sexual personhood also featured in many accounts. As might be expected, wider societal respect for one’s sexuality had most salience in the accounts of participants with minoritised sexual identities or who experienced stigmatized practices related to sex or sexual health (e.g. abortion). Those denied societal sexual respect sometimes minimized the importance of this negative regard by diminishing the social standing of those who held such views, describing them as “*outdated*” and “*old-fashioned*.” Others recounted actions taken to avoid situations where their sexual personhood would be disrespected. One woman, for example, stopped visiting pubs with her girlfriend, where she felt their romantic interactions attracted “*sleazy*” attention from men. Heterosexual participants generally placed less importance on their sexuality being accepted by wider society than those identifying as lesbian, gay, or bisexual; only a handful explicitly acknowledged that this stemmed from a taken-for-granted experience of social acceptance of their sexuality within heteronormative culture; for example: “*I guess being heterosexual, it’s obviously, I don’t worry about that* [feeling my sexuality is included and valued] *because it’s kind of like more I’m following the norm*” (Heterosexual man, aged 32, White, in a relationship). These findings suggest there may be a meaningful distinction between perceptions of respect for one’s sexual personhood, and respect for one’s sexual identity.

### Sexual Self-Esteem

Appraisals of oneself as a sexual being – both positive and negative – were a core feature within participants’ accounts of sexual wellbeing. Self-appraisals of specific dimensions of sexuality included feelings about one’s: sexual appeal; capacities to please and be pleased by partner/s; sexual orientation; and desires and sexual behavior. These descriptions resonate with a conceptualization of sexual self-esteem as multidimensional (Zeanah & Schwarz, 1996).

Within accounts marked by high degrees of sexual selfesteem, participants presented their sexual selves in a positive light, emphasizing that they were sexually confident, capable, comfortable, and deserving of a good sex life. Feeling in control of one’s sexual thoughts and desires, alongside congruence between one’s sexual desires and societal norms regarding sexual “morality,” allowed participants to feel content in their sexual selves; for instance: “*I wouldn’t have any kind of extreme desires about* … *I want to have sexual morals and I know the things that I like and I’m happy with that*” (Heterosexual man, aged 42, White, married). Positive self-assessments were also often grounded in an account of oneself as able to experience mutual sexual pleasure with a partner – described by some as the core purpose of sex. Those who saw their ability to have mutually pleasurable sex as fundamental to their sexual wellbeing contemplated the likely negative impact on their sexual self-esteem if they felt this were not the case: “*I’d feel my confidence would be knocked if I was with somebody who wasn’t, you know, who I didn’t please*” (Gay man, aged 22, White, in a relationship).

Within accounts suggestive of lower sexual self-esteem, participants conveyed a dislike of their sexual self, recounting feelings of shame, self-consciousness, dejection, or worthlessness. Their sexual stories presented them in a negative light, often situating themselves as unattractive, unable to provide pleasure to others, sexually deviant or in some way unable to meet desired sexual standards. One man, for instance, described his low sexual self-esteem in the context of his feeling that his performance of masculinity was viewed by other men as passive and non-threatening:

I’ve been kissed by [female] friends in front of their boyfriend and the boyfriend didn’t give a crap because […] I have been described as “harmless,” which I never know whether to take that well or not because it describes me as like, “oh he won’t do anything but at the same time he’s weak.” (Heterosexual man, aged 29, White, single)

Taken as a whole, accounts emphasized the malleability of sexual self-esteem and highlighted the range of factors shaping its variability over time. Participants pinpointed particular experiences that affected how they viewed themselves sexually, strengthening or detracting from their self-esteem, including weight change, postpartum bodies, aging, and illness; for example:

… feeling sexually attractive to others, I think that’s really important, it’s really important to me, and it’s something that’s kind of impacted since I’ve had the illness, because I’ve had physical changes to my body, to my face, and it makes me […] I used to be quite a confident person about my looks, and I’m not so much anymore. (Heterosexual man, aged 37, White, married)

For the majority, their sexual self-esteem appeared to be strongly shaped by external judgments, both positive and negative, from peers and in particular sexual partners. Multiple participants, for instance, cited a partner’s lack of interest in having sex or negative comments as having a detrimental influence on their sexual self-esteem:

I remember I was on top one time and he’s [former partner] like, “Oh why are you going so slow?” and that actually put me off for like having partners and that, like sometimes even now I don’t go on top of somebody and all that nowadays, like it’s in my mind […] that was one of them actually like just saying that and it just stuck in my mind thinking am I not doing it right, and it just, and I just, just pure anxiety of actually doing that now. (Bisexual woman, aged 25, White, single)

Beyond sexual partners, many participants placed importance on feeling sexually desirable to others, irrespective of their own relationship status or the gender or sexuality of those who might desire them; as one man noted: *“well, obviously I can’t be as good-looking as I was when I was 24, but in the general scheme of things, I think you’ve always got to think that somebody fancies you somewhere, and I think it’s quite important*” (Heterosexual man, aged 56, White, married). Women’s accounts in particular highlighted the gendered dimensions of normative physical attractiveness, drawing attention to the socio-sexual privileges that can be gained from meeting socially prescribed beauty standards:

… as a society I think, like even if you’re in a relationship you want to feel attractive to other people, not necessarily you want to attract them but you want people to think, oh yeah, she looks good, you know, so I think that’s quite important and as a woman I think you want… You want people to feel like, oh yeah, she’s good looking, so yeah. (Heterosexual woman, aged 29, White, married)

Accounts indicated a complex relationship between external appraisals of one’s desirability and sexual self-esteem. A minority of participants, for instance, discussed valuing themselves sexually or starting to accept themselves despite feeling they did not meet societal standards of desirability. They spoke about their efforts to strengthen their sense of sexual self-worth after feeling tired of the negative emotions and dissatisfying sexual experiences that stemmed from their low sexual self-esteem. This cognitive work included externalizing negative assessments made of them, reframing their sexual self-narrative and building their internal sense of worth (e.g. as someone deserving of a good sex life), for example:

I used to feel like that, like lack of confidence. But, in my eyes now, right, this is my motto. So, it’s my thing, I said, “well, you’re fat, and that’s it, so you might as well just make the most of it, and have a really good time” […] Don’t worry about your waist, or your this, or your that. Or what shape you’re in, just enjoy yourself and have good sex. (Heterosexual woman, aged 55, Black British, married)

As long as you feel good in yourself, don’t overthink it […] That could be age and maturity. The fact I’m not 25 anymore […] having that confidence in yourself is really, really, really important. For me, it’s not about feeling attractive to others, it’s about feeling attractive in myself. So, it’s never about what others think. (Heterosexual woman, aged 40, White, married)

Thus, these accounts highlight how feelings of sexual attractiveness within oneself, which encompass more than just self-perceived physical attractiveness or body image, can underpin sexual self-esteem.

### Comfort with Sexuality

The concept of comfort with sexuality – defined by us as an experience of ease within one’s contemplation, communication, and enactment of sexuality – pervaded participants’ accounts of sexual wellbeing. Its relevance was particularly evident within participants’ talk about bodily sensations and emotions during partnered sex. When participants spoke about comfort, they referenced feelings of openness, trust, acceptance, and relaxation with oneself and one’s sexual partner(s). For some, comfort was described as akin to mindfulness, that is, being able to escape the distraction of daily life through focus on “being in the moment”: “*I think that, for me, that’s part of what the whole sex gig is, to be in the moment and not worry about a leak in the kitchen*” (Heterosexual woman, aged 40, White, married); and “*it’s like the moment you share with somebody [*…*] you’re both in the moment and you’re both, it’s almost like a chance to not think about anything else but just you and your partner together*” (Heterosexual man, aged 32, White, cohabiting/in a relationship). While high degrees of comfort were described as facilitating pleasurable sexual experiences, accounts indicated that comfort was neither necessary for, nor an assurance of, pleasure.

Accounts of factors that generated a sense of comfort tended to fit into two broad categories. On the one hand, participants spoke about the importance of familiarity for deepening levels of comfort, which allowed people to enjoy vulnerable sexual moments. Familiarity could result from accumulating sexual experience over time as well as from experience within an ongoing relationship; as one woman (Lesbian, aged 20, White, cohabiting) said of her current sexual partner: *“She’s the first person I’ve felt fully comfortable to sleep with properly, do you know what I mean? I get butt naked and stuff whereas I wouldn’t have done that with anyone else.”* In contrast, participants also spoke about the significance of comfort in novel sexual moments, which could result in experiences of amplified excitement, eagerness, and pleasure. Importantly, some participants described comfort emanating from experiencing both novelty and familiarity with a sexual partner:

There was just so much passion and just everything with us [former long-term partner]. We just kind of like, we never had boundaries, well we had certain boundaries but we’re always kind of wanting to possibly try something new or think of something new and just think “oh could we, couldn’t we?” kind of thing. (Heterosexual woman, aged 24, Mixed ethnicity, single)

Challenges to sexual comfort were commonly described, including feelings of embarrassment, denial, unease, anxiety, physical pain, distractions, or worries. For many, lack of comfort manifested as an inability to enjoy sexual moments:

… if you don’t feel comfortable then you’re obviously like you’re going to be in panic mode like when it’s happening and so you won’t really be like concentrating on being relaxed and like enjoying it, you would be more like on edge […] when I was first having sex I used to have a real problem with this [being in the moment], like I used to be like having sex was like thinking about, like just having sex like thinking about anything, like my mind was just in a different place […] it took me a long time to like really like learn like how to like, because I just have the kind of mind where, unless the sex is really, really good, and like I’m really in the moment, I’m really, really quite, I’m just like my mind will just stray off. (Heterosexual man, aged 19, White, cohabiting)

While the majority of accounts of comfort related to ease during the enactment of sex, a minority of accounts referred to discomfort when contemplating elements of their sexuality. For these participants, all of whom disclosed experiences of trauma and rejection, discomfort stretched beyond specific sexual moments and involved a generalized sense of disquiet at their enjoyment of a sex life which they had come to view as detracting from their broader wellbeing. For instance, one woman (Heterosexual, aged 38, Asian British, single) reflected on her realization that she was “*attracted to danger*,” attributing this to her experiences as a young adult which had resulted in the normalization of abusive relationships: “*I had an epiphany, that’s what it is. I go for men that are dangerous. And it’s taken us* [meaning me] *nearly 40 years to realise this and it’s disgusting. It’s horrible. I just need to like, not*.” Maximising comfort required people to understand what they wanted for their sex lives. This was described as an understanding one grew into and which developed with the passage of time and experience, rather than being innate.

### Self-Determination in One’s Sexual Life

Although not labeled as such by our participants, the concept of self-determination – defined by us as the ability to negotiate autonomy of decisions over one’s sex life – resonated strongly within accounts of sexual wellbeing. Participants highlighted the importance of being able to maintain one’s boundaries, avoid one’s dislikes, and pursue one’s sexual wants and desires; for example:

… you always just need to have control and comfort with it, especially with your sexual thoughts and desires, it’s like you need to say to yourself, oh let’s say if you’re thinking to have threesome or anything, you should be able to say okay, well yeah, the thought for this, I’m actually very comfortable with it, I’m not afraid to say it or seek it. (Heterosexual woman, aged 24, Mixed ethnicity, single)

In particular, participants emphasized the relational nature of self-determination within their understandings of sexual wellbeing, often using terminology of “consent,” “control,” “boundaries,” and “mutuality”; as one participant said: “*you don’t want to be controlling but then you don’t want to be controlled so it’s got to be like a mutual kind of thing between each other”* (Bisexual man, aged 23, White, single). Participants expressed a view that maintaining self-determination went beyond the ability to simply agree or disagree to sexual activity, to also incorporate balancing control over *how* partnered sexual interactions unfolded, such as their context, pacing, or duration. Importantly, some participants (mainly heterosexual men) explicitly questioned the limits of self-determination and its relationship to sexual wellbeing in scenarios where an individual might pursue their own sexual interests to the detriment of a sexual partner:

… if it’s only for your sexual gratification, then the other person in the partnership is not getting that same thing out of it. You might say, oh, it’s my sexual wellbeing as well because I get real satisfaction, say, if you’re dominating in the whatever activities, then your other partner’s playing a passive role, then they feel like it’s a chore. That’s not sexual wellbeing for them, I think you’ve both got to be wanting to do whatever activities you do. (Heterosexual man, aged 56, White, married)

Thus, for some, sexual wellbeing was derived from being able to take into consideration what a partner wants, which might mean exercising self-control by not acting on one’s own desires. Accounts revealed multiple factors that could either strengthen or undermine self-determination, ranging from those at the individual and partner level through to those relating to wider social networks and culture. At an individual level, several participants talked about how, through growing experience, their ability to understand the types of sex and sexual partners they wanted had evolved with positive impacts on their sexual wellbeing; for example:

**Interviewer: … are there elements [of your sexual wellbeing] that have gotten better?**

I think it’s the ability to, erm, finding you sexually attractive to others, ‘cause when I did initially identify as gay I had pretty much, find anyone in the club and just kiss them. And then they’d invite me back and I’d says yes, let’s go for it, but … now I’ve gotten, I’ve taught me, well not taught myself, but I’ve found who I like, and who I would like to have, to see, possibly, see myself with. (Gay man, aged 25, White, single)

Factors limiting one’s sense of self-determination included sexual inexperience (which could inhibit the ability to understand and enact one’s boundaries and desires), and experiences which were either non-consensual or were perceived as having been more enjoyable for one partner than another. Additionally, multiple participants drew attention to the ways in which personal and community networks could shape self-determination. For example, one woman described the negative role that her ex-partner’s family played in enabling his multiple forms of abuse (financial, emotional, physical, and sexual) against her, with her ability to end the relationship complicated by her financial precarity. In contrast, another woman discussed the positive ways in which her Islamic faith and religious community informed her understanding of, and confidence in, communicating and enacting her sexual boundaries:

In Islam things like oral sex and things like that are sort of frowned upon, so the last thing I want is for someone to try and force that on me because I do not want to, I’ve already decided that is not what, I wouldn’t …

… **is that something you’d expect to like have a discussion about with the sexual partner?**

Well if it is like somebody who’s going to be on the same level of like religion as me then hopefully they’ll already understand but it may be worth a conversation just to kind of establish it. (Heterosexual woman, aged 20, Asian British, single)

Finally, as in other domains, accounts highlighted how self-appraisals of self-determination could evolve situationally and over time. For instance, one woman, now in her twenties, reflected on how she had once considered herself to have high self-determination for having multiple sexual relationships with older men as a teenager, but had subsequently come to view many of these experiences as non-consensual.

### Sexual Safety and Security

Our conceptualization of this domain is multi-layered; while safety is conceptualized as relating to feelings of limited threat in one’s *current* sex life, security is conceptualized as a longerterm sense of assurance about one’s sexual *future*. Feeling safe within one’s sexual life, which was commonly raised by participants early on in the interviews, referred to minimization of multiple potential risks, including those relating to STIs, unwanted pregnancy (where relevant), the emotional vulnerability of intimacy, unsolicited sexual attention (in person or digitally mediated), and the possibility of psychological, sexual, or physical threats or violence:

… you want to feel secure if you’re going to have sex with someone of course you want to feel secure and like safe and like you’re not in any danger […] You need to know when that person wants you to stop, like, to stop. (Bisexual woman, aged 25, White, in a relationship)

Accounts of safety as a fundamental component of sexual wellbeing were strongly gendered, with many more women than men expressing concerns about their physical safety and risk of sexual violence. While concerns about the possibility of emotional vulnerability may also have been felt by heterosexual men, it is possible that masculinity norms contributed to them feeling less able to voice these in the context of an interview. Concern about sexual violence and homophobia was also present in two gay men’s accounts, but rarely in those of heterosexual men:

… when you’re having hook-ups, as they call it, y’know, it is quite scary because you don’t know if you’re going to somebody’s home or they’re coming to yours and they know your address and then you have to sort of use your like your common sense in terms of like okay well that person’s discreet and they maybe have a partner or you know, whatever, but erm, really you don’t know, you don’t know, and you have to take sort of safety into account. (Gay man, aged 37, White, single)

Participants tended to reflect on feelings of safety in the present and near-future rather than a longer-term sense of security; as one woman noted, “*depending on where you are in your life, your sexual wellbeing, you’re not necessarily thinking about your future hugely, you’re thinking about the here and now and that as opposed to 5, 10 years down the line*” (Heterosexual woman, aged 40, White, married). Some participants’ accounts of safety referenced “not needing to worry” about risk in their sex life, which was often attributed to stability and trust within a current relationship, and belief in its longevity. For others, constant sensitization to the possibility of risk amplified concerns about safety, particularly among those with newer sexual partnerships, or who had present feelings of vulnerability within their sexual lives: *“like I would say it’s important to like keep it* [sexual risks] *in the back of your mind, if you know what I mean, like I wouldn’t just like not think about it”* (Heterosexual man, aged 19, White, cohabiting).

In the few accounts where participants reflected in greater depth on how current challenges within their sexual lives might play out in the longer term, they spoke of feeling confident that future sexual experiences would be positive, even if their present sex life was not ideal. Security in one’s sexual relationship was core to these understandings. One man, for instance, had recently been diagnosed with a serious illness which was affecting his energy levels, desire for sex and sexual self-esteem, with negative ramifications for his sexual relationship with his wife. Despite this, his account of the strength of their relationship and their adaptation to these changes underpinned his belief that his sex life would improve again over time: “*even though our sex life at the moment is not the best, I think it will be in the future* … *I’m not massively worried about it*” (Heterosexual man, aged 37, White, married). Thus, this account reflects the strong interpersonal element to security, where the quality of a relationship with a current partner can be an essential part of feeling secure about one’s sexual future in the face of adversity.

### Forgiveness of Past Sexual Experiences

Forgiveness was defined by us as the extent to which one has been able to make sense of, and move on from, past trauma and negative sexual experiences. Within the card sorting activity (see Table 3), the vast majority of participants rated statements relating to both self-forgiveness and moving on from negative past experiences and relationships as important for sexual wellbeing. Some participants (particularly heterosexual men) said the importance of forgiveness for sexual wellbeing applied to others, but not themselves, as they did not feel they had had negative or traumatic sexual experiences. For others, however, the nuanced challenges of forgiveness were evident within their accounts of personal efforts to process and move on from negative or challenging experiences within their sexual lives, including relationship breakdown, loss of trust, humiliating or non-consensual sexual experiences, abusive relationships, unplanned pregnancies, and homophobia. For those who had experienced severe and/or multiple trauma, this could be especially arduous. Yet despite struggling with feelings of residual hurt, anger, guilt, regret, or shame about past experience(s), participants recognized that patterns of negative thoughts toward oneself and/or others could reduce sexual satisfaction and inhibit opportunities for future intimacy, for example:

I think of experiences in the past where if you sort of dwell on things that’s happened, it can, it can harm you and your sexual wellbeing, you think about it, you put a barrier up […] And then you sort of, you don’t want to be intimate, and that’s quite important to move on, well, that’s happened, you can’t dwell on it, you can’t change it, let’s just sort of, move on from it. (Lesbian woman, aged 32, White, married)

As seen here, the language of “moving on” resonated strongly with participants and was commonly framed as a way to safeguard one’s sexual wellbeing against the toxic effects of dwelling on negative experiences. Across the sample, “moving on” was framed as a gradual process that requires effort over time. The factors that participants identified as enabling “moving on,” however, varied. Learning and growing from one’s mistakes was positioned as key to self-forgiveness by many. Some participants expressed self-compassion and relative ease in acknowledging past mistakes, for instance: “*We all make mistakes, and you’ve got to be able to, you know, be comfortable with yourself and say, “Look, mate, it was a mistake*” (Heterosexual man, aged 56, White, married). For others, however, it was more of a challenge, for example: “*I think that I should be able to* [forgive myself for past mistakes], *but I’m very, I put myself down quite a lot and think about stuff all the time*” (Bisexual woman, aged 25, White, single). Akin to the concept of decisional forgiveness (a behavioral intention to reduce one’s negative behavior), some described how “moving on” involved deliberate effort to disrupt and “let go” of patterns of negative thinking, or even trying to forget:

I put that down to like my ex really because obviously I wasn’t happy for so long, for like a year and I just need to kind, I’ve kind of let that go now. But it was, it used to build up and I used to think, “I’ll never want to have sex with anyone again.” Clearly you do but it’s just good to let it go […] I just feel so much better now, just because I’m trying to let it go. (Heterosexual woman, aged 24, White, single)

[Talking about a sexual image of himself and girlfriend being shared around school without their consent] I don’t know, I don’t really think I’m over it yet, but it’s, I don’t know, you kind of just learn to forget about it, I wouldn’t really say it’s really getting over it because I don’t think you get over stuff, I think you can just learn to cope with it better. (Heterosexual man, aged 19, White, in a relationship)

In contrast, several participants queried the benefit of forgetting or letting go, instead positioning “remembering” as key to their wellbeing. “Remembering” allowed them to see how they had learnt and grown from difficult experiences:

[talking about moving on from an abortion] I don’t feel like I need to let it go, I do think about it all the time. And I discuss it, I’m comfortable enough to talk to people about it. At the beginning I didn’t, I couldn’t even tell my mum […] do you know what the biggest thing was? I don’t want to forget about it and I don’t want to let go and I want to be able to think about my negative experiences because although it’s negative, it’s a positive now because my life’s still fine, I haven’t got a child that I can’t afford. (Heterosexual woman, aged 24, White, in a relationship)

### Resilience in One’s Sexual Experiences

Resilience was defined by us as the ability to cope, adapt, or experience positive outcomes in the face of risk and trauma. Although participants described a range of changes and challenges with ramifications for their sexuality and sex life (e.g. relationship breakdown, new relationships, partner bereavement, chronic health conditions, abortion, abuse, parenthood, and coming out), many struggled to discuss resilience in depth. Some commented that this was irrelevant for them, as they were in a long-term relationship – perhaps in part an artifact of the statement in the card sorting activity which emphasized adaptation to change (“*Feeling able to adapt to new sexual situations and/or partners*”). Others did not appear to resonate with the language of resilience, despite recounting responses to situations that might be understood by others within these terms.

In discussing what resilience within one’s sexual life might mean, participants mostly described sexual resilience as a response to a particular incident or situation, rather than an ability to proactively anticipate and tackle challenges. For some, resilience involved taking action to change a situation about which they were unhappy. For example, one woman described leaving an abusive partner and subsequently feeling stronger and more capable of resisting his sexual advances.

It was awful [abusive relationship and her ex going to prison], it really is, and that’s why I’ve toughened up a bit this time. I thought I’m not even going to give you the chance […] No matter how much I want me jiggy-jiggy. I just thought, no, I’ve got more self-respect for myself. I’m not prepared to put up with it. He had one chance and he’s blown it, so. (Heterosexual woman, aged 38, Asian British, single)

Connecting with themes in the Forgiveness domain regarding learning and growing from challenging situations, others viewed resilience as cognitively and emotionally coming to terms with a situation they could *not* change and restoring equilibrium; for example:

I’ve learnt a lot from it [his long-term relationship] coming out of it, I think after we’d split up, erm, I could kind of look at stuff in a different light and I’ve learnt a lot of lessons now that I would take into future relationships, so. (Heterosexual man, aged 22, White, single)

I think the only thing that’s changed is the fact that he’s gone [death of her husband] and that is it. I can’t really think further forward than that at the moment. But I think, yeah, if I find a partner and if I’m, great, great, fantastic, it’s time to move on. (Heterosexual woman, aged 46, British Indian, widowed)

We found instances of both individual assets and external resources that enabled people to maintain equilibrium, or even experience positive outcomes in the face of risk and trauma. While some spoke of their resilience as an inherent characteristic (e.g. “*I’m quite easy to get over things*,” Lesbian woman, aged 20), others described the important role that trusted and non-judgmental people played in their ability to deal with challenge and change over time. One woman, for instance, described the significant role of her mother and social workers in helping her heal from an assault as a child, allowing her to feel like she could ask for help and trust people. In contrast, others said they lacked people in their lives who could understand or relate to their situation, or help them cope with difficulties.

### Interaction Between the Domains

As demonstrated in previous sections, dimensions of each of the proposed domains resonated with participants’ accounts of their experiences and understandings of sexual wellbeing. Our data clearly demonstrated that domains do not operate in isolation, but rather interconnect to comprise sexual wellbeing as a multifaceted and complex construct. Through a detailed case analysis of 20 participants’ accounts, we mapped inter-linkages between the domains to support understanding of sexual wellbeing as a multidimensional construct. By looking across cases, we identified how dimensions of different domains can interact in ways that are *synergistic* or *antagonistic*. We now present two case studies to illustrate how these dynamic interactions (both synergistic and antagonistic) between domains of sexual wellbeing play out in the context of individual lives. For instance, the ability to forgive oneself for regretted elements of past sexual experiences can be strengthened through social support (a synergistic interaction between domains of Forgiveness and Resilience illustrated in Case Study 1). In contrast, sexual experiences that may be characterized by a high degree of ease and satisfaction in the moment may nevertheless generate feelings of shame after-ward if they diverge from what one perceives is socially acceptable (an antagonistic interaction between domains of Comfort, Self-Esteem and Respect illustrated in Case Study 2). Our inclusion of these case studies also offers further illustration of how sexual wellbeing is dynamically shaped through the interplay of social and structural influences over time, and is experienced as an ongoing *process* that is worked on, rather than a definitive state that one can achieve.

### Case Study 1

Danielle is a heterosexual woman in her mid-20s who first had sex in her mid-teens and has since had several sexual partners. In describing her early sexual experiences, Danielle spoke about feelings of low **self-esteem**, including self-consciousness about her weight in a culture that valued thinness, and a lack of confidence in navigating her sexual wishes (**self-determination**). These early experiences of low self-esteem and low self-determination detracted from her ability to enjoy sex and be “in the moment” (**comfort**):

An aspect earlier in life was probably like “what are they [her sexual partners] thinking, what if they’re thinking, comparing me to other girls,” and like you know when you’re younger and you’re not experienced, that’s what you do think and a lot of time you think “*do they want me to take control, do they want to take control*?,” *so yeah you’re always thinking* …

A turning point in Danielle’s sexual wellbeing was her experience of an unplanned pregnancy and subsequent abortion in her late teens. At first, this experience took a heavy toll on her wellbeing. She felt out of control and unsupported by her sexual partner, who accused her of lying and blocked her on social media. This led to a difficult period in which she drank and partied in order to cope by “*keeping busy*” so she “*didn’t have to think about it*.” Despite this initial difficulty, her unplanned pregnancy and subsequent abortion motivated her to enact positive changes. By the time of the interview, she felt she had processed her experience, and found “silver linings” in it. She had an implant fitted at the time of the abortion which imbued her with a greater sense of control and **security** over her reproductive future. Additionally, the experience gave her the resolve to avoid sexual situations and relationships where she felt similarly out of control, and a belief in her ability to uphold these boundaries (**self-determination**). In contrast to her early efforts to avoid thinking about her abortion, she now talked about not wanting to “*let it go*,” but rather wanting to hold it in her mind because she had learnt valuable lessons from it: *“I mean I’m not saying it was a good experience, it was an awful experience, but now I can think about it positively and I can, I still think about it”*. Key to her capacity to reframe this challenging experience in terms of its positive impact (**self-forgiveness)** were the non-judgmental and supportive responses from her mother and grandmother who she was able to talk to about her abortion and who validated her decisions (**resilience**).

Danielle spoke about her sexual wellbeing as something that had grown within the context of her current supportive longerterm relationship. The high degree of mutual trust and **respect** she shared with her partner, alongside his efforts to lessen her ongoing insecurities about her sexual desirability (**self-esteem**) led her to experience a more passionate and enjoyable sex life (**comfort**). Yet while Danielle was clear that she saw sexual wellbeing as broader than momentary pleasure and satisfaction, she appeared to struggle with accepting fluctuations in her desire and interest in sex – perhaps partly because this deviates from dominant social expectations that a good sex life is characterized by wanting and having frequent sex. As such, Danielle’s account of her current sex life conveyed her sexual wellbeing as a work-in-progress.

### Case Study 2

Steve is a single gay man in his late thirties who described struggling intensely with his sexuality throughout his life. Steve spoke about the lack of **respect** for his sexual personhood that he experienced growing up in the 1990s under Section 28, where “gay” was routinely used as an insult and he lacked gay role models and social contacts: *“I didn’t know anybody that was gay, I wasn’t friends with anybody that was gay”*. These experiences of societal disregard of his sexuality detracted from his **self-esteem**, as Steve internalized negative messaging. He felt ashamed of his and others’ gayness, mentioning how others have called him “*quite a homophobic gay.”* This lack of **self-respect** shaped his **sexual comfort**; he pursued shortlived, secretive sexual encounters with other closeted men to avoid risking his social circle’s regard for him. While he found enjoyment, pleasure, and a high level of **comfort** during these sexual experiences, as lies and secrecy became entrenched, his **self-esteem** further weakened, and he began to feel ashamed about these sexual encounters, outside of the sexual moments themselves. His **low sense of self-esteem** undermined his **self-determination** to ensure his own safety within sexual encounters, including meeting sexual partners in situations and places in which he did not feel physically or psychologically safe. Feeling unworthy and undesirable also impeded his capacity to pursue relationships in which he would feel loved, valued, and appreciated. For years, he remained in a turbulent and unhealthy relationship with a married man who was not open about his attraction to men. Steve’s **resilience** was weakened, as hiding his sexual identity socially isolated him and detracted from his ability to ask others for support:

I wish that probably [I] had somebody to speak to when I couldn’t come out, when I was seeing somebody that I shouldn’t have been seeing and you know, I was embarrassed and ashamed and anxious…

After the end of that tumultuous relationship, however, Steve felt his sexual wellbeing somewhat improve. By his late twenties, and in the context of shifting socio-cultural visibility and acceptance regarding LGBTQ+ identities and relationships, he had progressively come to accept his sexuality and started to communicate it to selected others. After coming out, he described feeling more **comfortable** desiring relationships with other openly gay men. Nevertheless, at the time of interview, he had still not **forgiven** himself for his relationship with a married man and was still somewhat **uncomfortable** referring to himself as gay, particularly in professional settings. The ongoing emotional weight of his **unforgiven** past and his lack of pride and **self-esteem** in his sexual orientation detracted from feelings of longer-term **security**, making him feel hopeless about his sexual and romantic future.

## Discussion

Based on accounts from a diverse sample of adults, this study provides empirical support for conceptualizing sexual wellbeing as a multidimensional and dynamic construct that is distinct from sexual health and sexual satisfaction. Specifically, we conceptualize sexual wellbeing as comprising emotions and cognitions regarding one’s sexuality and sexual life, which include feeling safe, respected, comfortable, confident, autonomous, secure about one’s sexual future, and able to work through change, challenge, and negative past experiences. Sexual wellbeing is shaped through social and structural influences, including individuals’ interactions with sexual partners, other social connections, and wider environments, including education, health, political and legal systems, and intersecting systems of social inequality.

In the following discussion, we reflect on novel conceptual insights generated through this qualitative study which add nuance to our initial conceptualization of sexual wellbeing. We also outline questions to pursue within a research agenda focused on deepening understanding of sexual wellbeing.

### Conceptual Insights

#### Sexual Wellbeing is Conceptually Distinct From, Yet Related To, Sexual Satisfaction and Pleasure

While sexual satisfaction and pleasure were central within participants’ talk about what it means to have a good sex life, their accounts clearly distinguished these facets from the concept of sexual wellbeing. In essence, sexual wellbeing was conveyed as a summation of one’s broader experiences and expectations related to sexuality that *enable* pleasurable sexual experiences and a satisfying sex life. Although our participants often appeared to use sexual satisfaction and sexual pleasure synonymously, these are established in sexology literature as distinct constructs (Pascoal et al., 2016; Reis et al., 2021; Werner et al., 2023). Sexual satisfaction is commonly conceptualized as a global evaluation of the positive and negative dimensions of one’s sex life, including satisfaction with sexual activities and sexual relationships (Lawrance & Byers, 1995; Park & MacDonald, 2022). Sexual pleasure, by contrast, is typically characterized as a more transitory experience of satisfaction derived from sexual activity (solo or partnered), with multi-faceted conceptualizations encompassing interplay between cognitive, emotional, physical, and sensory elements. Some theorists have further differentiated between state-like sexual pleasure (“feeling good” during sexual activities) and trait-like sexual pleasure (the *tendency* to enjoy sexual activities) (Werner et al., 2023). Despite these nuanced differences, conceptualization of both sexual satisfaction and sexual pleasure primarily focuses on *experiences of sexual activity itself*, thus contrasting with how participants in this study viewed sexual wellbeing as extending *beyond experiences of sex*. Thus, our participants’ accounts support conceptualization of sexual wellbeing, sexual pleasure, and sexual satisfaction as distinct yet interconnected aspects within a comprehensive public health perspective on sexuality (Mitchell et al., 2021).

This distinction also chimes with the recent multifaceted definition of sexual pleasure endorsed by the World Association for Sexual Health (Ford et al., 2019), which delineates between sexual pleasure (defined as “physical and/or psychological satisfaction and enjoyment derived from shared or solitary erotic experiences”) and its “enabling factors” (e.g. self-determination, safety, consent) which provide a route “for pleasure to contribute to sexual health and well-being.” A key nuance offered by our conceptualization is that rather than positioning these “enabling factors” as mechanisms through which pleasure *contributes to* wellbeing, these factors are *constitutive of* sexual wellbeing itself. Thus, we assert that facets of sexual wellbeing – such as having sexual self-esteem, feeling respected, safe, and resilient – set the stage for pleasurable sexual experiences. While sexual pleasure and sexual wellbeing are conceptually distinct, they are nevertheless highly inter-related such that the accumulation of these enabling factors in relation to one’s sexuality (e.g. self-determination, safety, respect) will facilitate the attainment of pleasure – and conversely, constraints on these will inhibit the likelihood of pleasurable experiences. As such, both sexual wellbeing and sexual pleasure can be understood as integral to a positive and satisfying sexual life. With regard to measurement, the complex interconnections between sexual pleasure and wellbeing conveyed by our participants supported a subsequent decision to empirically test inclusion of an item on sexual pleasure within the measure of sexual wellbeing (Mitchell et al., 2023).

Within research agendas focused on positive aspects of sexuality, clearer understanding of connections between sexual wellbeing and sexual pleasure may help further elucidate the sociostructural inequities that give rise to “pleasure gaps” – the well-documented gender differences between cisgender women and men in enjoyment of partnered heterosex, including discrepancies in orgasm (Mahar et al., 2020; Wetzel & Sanchez, 2022). In their recent essay, Laan et al. (2021) distinguished between the *capacity* to experience sexual pleasure and *opportunities to do so*, which are shaped by “societal pressures which facilitate or penalize attainment and expression of pleasure.” Contrary to popular discourse that women are somehow less biologically or psychologically capable of experiencing pleasure, rather, they argue that sociocultural factors present a longer list of obstacles to women experiencing pleasure within partnered heterosex than men, including women’s greater likelihood of receiving social disapproval for having sex outwith a relationship, greater concern of threat of sexual violence, and gendered sexual scripts that prioritize men’s pleasure through focus on vaginal-penis intercourse, and male orgasm (see also Willis et al., 2018). Conceptualization of sexual wellbeing as a construct encompassing domains such as sexual safety, self-determination, and sexual respect may provide a productive route forward for measurement which enables analytic attention to gendered differences in these socioculturally shaped realms of experience that underpin “pleasure gaps” in heterosex.

#### Sexual Wellbeing is Multidimensional and Dynamic

Our findings support the emerging consensus that sexual wellbeing is a multidimensional construct (Lorimer et al., 2019; Sundgren et al., 2022), while also contributing several areas of nuance to this multidimensionality. First, participants clarified dimensions considered most important within specific domains (see Table 2). Within the self-esteem domain, for instance, high importance was placed on dimensions such as being able to give and receive sexual pleasure with partners, feeling sexually desirable to others, and feeling sexually attractive within oneself, but low importance was given to feeling one has opportunities to have sexual experiences.

Second, accounts clarified that all seven domains were relevant within participants’ characterizations of sexual wellbeing in general, but the personal salience of certain dimensions varied between individuals. In other words, some individuals described certain domains (or dimensions of domains) as highly relevant for the sexual wellbeing of others, but less so for themselves. In general, these dimensions were most salient for those with expectations or experiences of adversity relevant to that domain. For instance, while heterosexual men recognized the importance of sexual safety and security, this domain was most personally salient in accounts of women (irrespective of sexual orientation) and gay men, who articulated a greater sense of threat relating to sexual and physical violence. The uneven patterning of coercion and violence, and salience of domains of forgiveness, respect, and resilience among those who had encountered adversity (particularly women and people with minoritised sexual identities), illustrated how inequalities in sexual wellbeing are shaped through broader intersecting systems of inequality.

A third area of insight regarding the multidimensionality of sexual wellbeing is the nature of the dynamic interactions between domains. Participants’ accounts indicated how domains can interact in ways that are *synergistic* (e.g. how feeling respected by a sexual partner can lessen insecurities and boost sexual self-esteem) or *antagonistic* (e.g. how investment in maintaining positive regard of one’s sexual personhood by significant others, such as a long-term partner, may present constraints to pursuing particular desired sexual activities). As such, accounts revealed how sexual wellbeing as a construct allows attention to potentially contradictory – or *antagonistic –* dimensions of sexual experience in ways that “sexual health” seldom attends to.

With respect to connections between domains, an area for future work is the interconnection between Resilience and Forgiveness. Among the participants in this study, overlapping accounts relating to concepts of “learning from one’s mistakes” and “moving on” raise questions about whether these are best conceptualized as distinct domains, or rather sub-dimensions of a different overarching domain. Further work could explore how these domains are empirically connected (e.g. is resilience a prerequisite for forgiveness, for instance?). Moreover, how are processes relating to these domains best articulated and deployed in public health, clinical, and therapeutic approaches to supporting sexual wellbeing?

Fourth, participants conveyed a sense of sexual wellbeing as an ongoing process that fluctuates over time – it is constant work-in-progress, rather than an enduring state one can achieve. Elements of both *biographical* time (over the life course) and *historical* time (wider frames of external events, environments and political landscapes (Adam et al., 2008) were evident within participants’ accounts, reflecting the dynamic interplay between individual actions to protect one’s sexual wellbeing over time and evolving sociocultural factors that promote or inhibit sexual wellbeing. For instance, narratives of sociocultural change permeated participants’ accounts of their life course transitions (e.g. cohabitation, parenthood, aging) that had led to learning, self-development, and growth, with consequences for sexual wellbeing, as well as shifts in their appraisals of past experiences (e.g. coming to view past experiences more positively or negatively with the passage of time) and expectations for one’s sexual future. Major societal shifts that infused participants’ talk included more expansive public understandings of sexual coercion and violence, more positive representations of aging and sexuality, increasing awareness and acceptance of diversity in sexual orientation, and changing gender norms. Sensitization to these *temporal* dimensions helps focus attention on the broader social conditions through which sexual wellbeing might be strengthened or jeopardized through social change, not only individually but also collectively. Moreover, participants’ characterization of sexual wellbeing as subject to intrapersonal monitoring and review also confirms the importance of its conceptualization as relating to both past experiences and near-future expectations.

#### Sexual Wellbeing is Socially and Structurally Influenced

Participants’ accounts in this study clarify that social and structural factors are integral to how sexual wellbeing is conceptualized by the general public – a characterization not currently well reflected in measurement of sexual wellbeing, which is largely focused on cognitive-affective dimensions at the individual level (Lorimer et al., 2019, 2023; Sundgren et al., 2022). A particular contribution offered by this study is foregrounding of the important role that social relationships (beyond those with sexual partners) can play in strengthening or undermining one’s sense of sexual wellbeing. Examples include having social support to cope with challenge and change in one’s sex life (Resilience), feeling one’s authentic sexual personhood is valued and affirmed by friends and family (Respect), or the deleterious impact of rigid gender norms within a peer group on one’s sense of sexual attractiveness (Self-esteem). This finding is important in the context of Lorimer and colleagues’ review of sexual wellbeing measurement (Lorimer et al., 2019), where the vast majority of dimensions categorized as interpersonal (self in relation to another) pertain to sexual partners (e.g. dimensions such as relationship satisfaction, partnership communication, relationship goals, and experience of violence within one’s relationship), and not to other significant relationships within one’s interpersonal networks, such as those with family members and friends.

Beyond the known others within an individual’s social network, participants also described features of the wider structural environment that influenced sexual wellbeing, including community and societal norms, and legal and policy contexts. With respect to norms, while participants emphasized the importance of how they were viewed by those closest to them (“significant others”), accounts also demonstrated the importance of the “generalized other” in shaping self-appraisals relating to the domains. For instance, perceptions of norms relating to gender and sexual orientation can impact upon sexual wellbeing, either positively (e.g. providing a positive framework of values to draw from) or negatively (e.g. internalization of societal homophobia, or normalization of gender-based violence fueling low expectations for positive sexual experiences). Policy and legal contexts, of course, both shape, and are shaped by, societal norms, and accounts indicated (both explicitly and implicitly) a variety of policies with relevance to sexual wellbeing.

Taken in combination, participants’ perspectives support conceptualization of interpersonal and structural elements as central to the experience of sexual wellbeing, rather than peripheral. Understanding how social and structural systems enable, or fail to enable, sexual wellbeing connects strongly with recent calls for greater attention to “erotic equity” (Higgins et al., 2022) and is an important lens for a future research agenda concerned both with establishing the patterning of inequities in sexual wellbeing at a population level, and developing community-based and structural approaches to improving sexual wellbeing, alongside those focused at individual or partnership levels. As such, our concept of sexual wellbeing is embedded with the concept of sexual justice as a space for recognition and redress of inequities.

As Lorimer et al. (2023, p. 5) asserted, it is one thing to acknowledge wider socio-structural influences and another to capture them in an evaluative environment. Sociological work suggests that it is through affective dimensions that the significance of structure is made available (Denzin, 1990). The task for those focused on measurement of sexual wellbeing at the level of individuals is thus to develop suitable items that can attend to challenges of measuring interpersonal and sociocultural elements from a personal perspective, and also identify factors that enable people to convert aspects of social and structural environments into resources. Alongside efforts to measure sexual wellbeing among individuals, development of indicators to measure and track sexual wellbeing at the structural level could provide vital progress. For instance, assessing international variability between countries as contexts shaping sexual wellbeing might include indicators looking at legislation and policy prioritization relating to affirming sexual and gender diversity and protecting sexual and gender minorities, prevention of gender-based violence, and destigmatising STIs and HIV. Indexes might also track the availability of, and equitable access to, comprehensive, medically accurate, and gender-inclusive sexuality education, and to high-quality sexual and reproductive health services, including clinical care relating to PrEP, contraception, abortion, and menopause.

#### Strengths and Limitations

This study had several strengths. First, the qualitative study design enabled in-depth insight on accounts of sexual wellbeing, thereby providing an empirical basis with which to interrogate a conceptual framework for sexual wellbeing generated through researchers’ engagement with wide-ranging literature. This methodological approach enhances our conceptualization of sexual wellbeing by offering novel insights regarding the dimensions of domains articulated as most important, interconnections between domains, and fluctuating salience for individuals over time. Second, by drawing on a sample of participants diverse in terms of age, gender, sexual orientation, relationship status, socio-economic status, and geographic location within Britain, this study allows us to explore how the conceptual framework resonates across different population groups, including how the personal salience of domains varied between individuals. Third, the research team involved in interviewing and analysis included experienced researchers with different social identities (e.g. in terms of age, gender, sexuality, nationality, relationship status), thus strengthening the process of data generation and leading to richer interpretation of meaning.

There were also several limitations to this study. First, accounts generated during in-person interviews might be subject to social desirability bias. To mitigate this risk, interviews were conducted by researchers experienced in conducting interviews about sexuality and establishing rapport with participants, but it is possible that participants limited articulation of feelings and experiences they viewed as non-normative. Second, as sexual wellbeing was a secondary focus of the interview for half of the sample, these accounts were briefer than for the half of interviewees where sexual wellbeing was the primary focus of the interviews. Nevertheless, all participants discussed what sexual wellbeing meant to them, and all then completed the card-sorting activity. Our synthesis (including illustrative quotations) is based on the analysis of all 40 interviews, with the exception of our case analysis, which was based on 20 interviews. Third, although participants’ social and generational locations varied considerably, their views and experiences of sexual wellbeing inevitably reflect specificities of the socio-historical context of Britain, including longstanding availability of nationalized health care (including legal abortion), widespread provision of school-based sex education (albeit of varying quality), and, more recently, legal protection against discrimination based on a range of characteristics, including sexual orientation and gender reassignment (Equality Act, 2010). We hope that the salience of the conceptual framework can be assessed in diverse geo-cultural contexts to support better understanding of its relevance across different legal and sociocultural environments. Fourth, although our sample included people of different ethnicities and living in areas across a spectrum of deprivation, few participants spontaneously talked about sexual wellbeing in the context of ethnicity or social class, and we did not systematically probe in depth on this. Exploring how experiences of sexual wellbeing vary by intersecting axes of inequality, including ethnicity, social class, (dis)ability, neurodiversity, religion, citizenship status, and so on is therefore an important direction for further research, especially in the context of pervasive social inequalities. Moreover, while the conceptual framework for sexual wellbeing was designed to be relevant for all regardless of gender or sexual identity, attraction, or behavior, none of the participants expressed an asexual identity or non-binary gender identity. Careful further work is therefore needed to explore the extent to which this framework resonates with gender expansive and asexual people.

For those wishing to understand and address pervasive inequities relating to sexuality, attention to sexual wellbeing as a distinct construct offers a route forward. By contributing novel empirical evidence on meanings of sexual wellbeing among the general population in Britain, this paper provides insight into how sexual wellbeing is conceptualized, which has informed the development of a new measure of sexual wellbeing designed for a population survey (the Natsal-SW) (Mitchell et al., 2023). We hope these insights can inform the development of a more nuanced public health policy agenda that evolves beyond a primary focus on risk-taking and adverse sexual health outcomes to also integrate sexual wellbeing as an essential concept.

## Supplementary Material

Supplemental data for this article can be accessed online at https://doi.org/10.1080/00224499.2024.2326933

File 1

Table 1

## Figures and Tables

**Table 1:** Participant characteristics.

Participant characteristics*	Number of participants *(n* = 40)
**Gender**	
Women	20
Men	20
**Age group**	
18–24	16
25–39	16
40–64	8
**Sexual orientation**	
Bisexual	4
Gay	4
Lesbian	2
Heterosexual/straight	30
**Ethnicity**	
Asian, Asian British	4
Black African, Black British	2
Mixed ethnicity	1
White British	33
**Current relationship status**	
In a relationship	10
Married	12
Single	18
**Area within Britain**	
Midlands (England)	5
North England	10
South England	10
Scotland	10
Wales	5
**Area level deprivation (measured by postcode)**	
Above median	20
Below median	20

* Derived from participants’ responses to pre-defined categories used by market research agency during recruitment process.

**Table 2:** Proposed domains of sexual wellbeing: insights generated through qualitative interviews (*n* = 40).

Domain	Researchers’ initial definition of domain	Operationalized statements used in card-sorting activity during qualitative interviews	Selected examples of learning from qualitative interviews to inform measure development (Natsal-SW)
Sexual respect	One’s perception of positive regard by others for one’s sexual personhood.	"Feeling my sexuality is included and/or valued by others" "Feeling the people around me share the same values in terms of sexuality"	Participant terminology: acceptance Greater importance placed by participants on feeling respected by significant others (e.g. sexual partners, friends, family members) over the generalized other Feeling respected by wider society had greatest salience in accounts of LGB participants and those reporting stigmatized experiences (e.g. abortion)
Sexual self-esteem	Positive appraisals of oneself as a sexual being. This includes self-assessments of specific dimensions of sexuality such as sexual appeal, the capacity to please and be pleased by partner/s, congruence between sexual thoughts, desires and morals, and control over sexual thoughts and desires.	"Feeling sexually attractive to others" "Feeling able to please sexual partners" "Feeling able to be pleased by sexual partners" "Feeling 1 have opportunities to have sexual experiences" "Feeling my sexual thoughts and desires are in line with my sexual morals" "Feeling in control of my sexual thoughts and desires"	Participant terminology: confidence (high self-esteem) and shame, lack of control (low self-esteem) High importance placed on being able to give and receive sexual pleasure with partners, feeling sexually desirable to others, and feeling sexually attractive within oneself Sexual self-esteem can be affected (positively or negatively) by physical change to body, external judgments, and internal cognitive work to reframe how one thinks of one’s sexual self
Comfort with sexuality	One’s experience of ease in contemplation, communication, and enactments of sexuality and sex.	"Feeling able to be ‘in the moment’ with sexual partners and not worry about other things" "Feeling a sense of peace/comfort with who 1 am sexually"	Participant terminology: being in the moment, focus (high comfort); distracted, uncomfortable (low comfort) High importance placed by all on comfort during enactments of sex (e.g. being able to “be in the moment” and “focus” during sex) Comfort with one’s sexual self usually interpreted by participants as comfort about sexual orientation
Self-determination in one’s sex life	One’s perceived ability to negotiate autonomy over one’s sex life. This includes a person’s capacity to experience their sexual wants and desires, while also maintaining their boundaries, and avoiding their dislikes.	"Feeling a sense of control/ability to have sex at times and places that feel comfortable" "Feeling a sense of control over the sorts of sex 1 want to have" "Feeling a sense of control/ability to choose who 1 have sex with" "Feeling able to choose sexual partners that 1 want, without pressure from others"	Participant terminology: mutual, consent, control, boundaries Emphasis on relational understandings of self-determination within partnered sex One’s sense of self-determination can be either strengthened or undermined by personal and community networks
Sexual safety and security	The extent to which one experiences feelings of limited threat when considering their sexuality and sex life in the present, and assurance about their sexual future.	"Not feeling a need to be vigilant about sexual risks" "Feeling a general sense of security about future sexual experiences (i.e. things will most likely be OK)"	Participant terminology: feeling safe, vulnerable, worried Emphasis on threats of sexual and physical violence, with greatest salience in accounts of women (irrespective of sexual orientation) and gay men Greater focus on "feeling safe" in the present and near-future rather than far-future
Forgiveness of past sexual experiences	The extent to which one has been able to make sense of, and move on from, past trauma and negative sexual experiences.	"Feeling able to forgive myself for past mistakes" "Feeling able to ’move on’ from negative past experiences" "Feeling able to "let go" of negative thoughts, experiences or relationships"	Participant terminology: moving on, learning from, letting go, coming to terms with High importance placed on forgiveness for past sexual experiences as key to sexual wellbeing in general, but some (particularly heterosexual/straight men) do not feel this applies to their own experiences Greatest salience in accounts of those reporting negative or traumatic sexual experiences (e.g. breaches to agreements of exclusivity within a sexual relationship, sexual humiliation, violence)
Resilience in relation to sexual experiences	One’s resources and ability to cope, adapt, or even experience positive outcomes in the face of risk and trauma.	"Feeling able to adapt to new sexual situations and/or partners"	Participant terminology: coping Framing of resilience as adaptation to new situations/partners resonated less with people in long-term relationships Participant emphasis on importance of social resources (e.g. trusted individuals) to cope with challenge and change.

**Table 3:** Participant rankings of statements in card sorting activity.

Domain	Statement on card	Important*	Not important**
Respect	Feeling your sexuality is included and/or valued by others	30	10
Respect	Feeling the people around me share the same values in terms of sexuality	15	25
Self-esteem	Feeling able to be pleased by sexual partners	38	1
Self-esteem	Feeling sexually attractive to others	37	3
Self-esteem	Feeling able to please sexual partners	36	3
Self-esteem	Feeling my sexual thoughts and desires are in line with my sexual morals	30	9
Self-esteem	Feeling in control of my sexual thoughts and desires	30	9
Self-esteem	Feeling I have opportunities to have sexual experiences	27	13
Comfort	Feeling able to be “in the moment” with sexual partners and not worry about other things	37	3
Comfort	Sense of peace/comfort with who I am sexually	34	5
Self-determination	Feeling a sense of control over the sorts of sex I want to have	37	3
Self-determination	Feeling a sense of control/ability to have sex at times and places that feel comfortable	36	4
Self-determination	Feeling a sense of control/ability to choose who I have sex with	35	4
Self-determination	Feeling able to choose sexual partners that I want, without pressure from others	30	10
Security	Feeling a general sense of security about future sexual experiences (i.e. things will most likely be OK)	33	6
Security	Not feeling a need to be vigilant about sexual risks	25	15
Forgiveness	Feeling able to “let go” of negative thoughts, experiences or relationships	36	4
Forgiveness	Feeling able to forgive myself for past mistakes	34	6
Forgiveness	Feeling able to “move on” from negative past experiences or relationships	33	6
Resilience	Feeling able to adapt to new sexual situations and/or partners	34	6

* Important category includes cards placed in “Very” or “Quite” important pile.

** Not important category includes cards placed in “Not that important” pile only.

NB Columns do not tally to 40 in all cases as some cards were unclassified in a small number of instances.

## Footnotes

**Disclosure Statement**
No potential conflict of interest was reported by the authors.
