# Supplementary material for: Conceptualizing Sexual Wellbeing: A Qualitative Investigation to Inform Development of a Measure (Natsal-SW)
Source: J Sex Res. Author manuscript; Available in PMC 2026 Nov 7. (PMC7619709; doi:10.1080/00224499.2024.2326933)
Supplement: Supplementary file 1 [file EMS220009-supplement-File_1.doc]

**Topic Guide for Sexual Wellbeing interviews (Natsal-4)**

**INTRODUCTION**

- Ensure participant has read the Participant Information Sheet
- Re-explain the study purpose and request permission for digital recording.
- Reiterate data confidentiality
- Offer to answer any questions (repeat this offer at the end of the interview as well).
- Complete consent form and give one copy to participant.
- Check we have recruitment demographic information
- Signpost structure of interview

**BACKGROUND**

a) In your own words, can you tell me a little bit about yourself? [Probe: where grew up, where live, family life, current employment/study, friends]
b) Please tell me how you identify in terms of your gender and sexual identity.
c) Please tell me about your current or most recent sexual or intimate relationship. [Probe: Who? How did you meet them? How would you describe this relationship?]
d) Please tell me about your previous sexual or intimate relationships. [Probe: Same or different sex partners? About how many partners?]

**SEXUAL WELLBEING**

**PART 1: Meanings of sexual wellbeing**

a) How do you feel about your sex life?

Open response, and then probe:

- What’s a good sex life to you?
- Are there times when your sex life has been better/worse?
- What has made a difference to any changes?

b) As you know, we’re interested in talking about sexual wellbeing, but before we get onto that, I just wondered if you could say a little bit about what wellbeing in general means to you?

Open response, and then probe:

- What does it mean to be or feel well/not?
- Relative importance of physical health, mental health, social wellbeing.

c) What does sexual wellbeing mean to you?

Open response, and then probe:

- What sorts of things would you consider if asked to think about your own sexual wellbeing?
- What sorts of things would you NOT include?
- Is wellbeing similar/different to sexual satisfaction or pleasure? If yes/no, how and why/not?
- What would it mean to not have sexual wellbeing? (open responses, and then probe pain, upsetting / scary experiences, lack of confidence, lack of self-esteem, partner factors, etc? [*interviewer to use blank cards to write in participant’s meanings on cards for Part* 2]*.*

d) Has how you think about sexual wellbeing at this point in your life changed over the course of your life?

Open response, and then probe:

- Any reasons for change (or lack of) in thinking now compared to then?

e) Has your own sexual wellbeing changed over the course of your life? Why/not?

Open response, and then prompt/probe:

- Can you think of a time when you had good sexual wellbeing?
- What was it about that time?
- Was this influenced by anything in particular?

**PART 2: Exploring relative importance of different dimensions of domains**

As part of our process for designing survey questions on sexual wellbeing, we are trying to understand how important different experiences and feelings might be to people in how they think about sexual wellbeing.

[*Interviewer to get out materials: 20 cards with statements + add in any that participant has added in PART ONE*]

Exercise: Please could you take a look at these cards, and sort them into three piles, according to the following statement:

**“*To say I had sexual wellbeing the following is... 1) very important, 2) quite important, 3) not that important.”***

*As participant is sorting cards, probe*:

- Rationale for placement of each card in each pile, including any they struggle to categorise.
- Ask them to talk through particular cards and put statements into their own words.

*At end, interviewer to take photo of the three piles*

a. Are there any things that doing the exercise has made you think about in relation to sexual wellbeing?
b. Are there any other things that you want to add?

End interview.
