# Supplementary material for: Conceptualizing Sexual Wellbeing: A Qualitative Investigation to Inform Development of a Measure (Natsal-SW)
Source: J Sex Res. Author manuscript; Available in PMC 2026 Nov 7. (PMC7619709; doi:10.1080/00224499.2024.2326933)
Supplement: Supplementary file 2 [file EMS220009-supplement-Table_1.doc]

**Table: Example codes from open coding on meanings of sexual wellbeing**

Code name

**Meanings of SW**

A good sex life

Balance

Being experienced

Being in love

Being proactive

Boundaries

Burdened

Circumstantial and environmental factors

Closeness to partner

Comfort with your body

Communication

Compromise

Connection

Confidence

Congruence

Consent

Excitement

Expectations

Experimentation

Feeling grateful

Feelings, no feelings

Freedom

Frequency

‘Happen naturally’

Happiness in a relationship

Honesty

Importance of sex

Interpersonal SW

Intimacy

'Knowing what you want (and what you don't)'

‘Knowing yourself’

Libido

Love-lust split

‘Making an effort’

Not doing things you don't feel comfortable with

Openness with partner

Pleasure

Power imbalance between partners

Protection from pregnancy

Protection from STIs

Relationship goals

Regret

Safety

Satisfaction

Self-control

Self-respect

Sexual anxiety

Similarity or compatibility between partners

Spontaneous VS responsive desire

Trust

‘Well enough for a relationship’
